# Analysis of real-time PCR *Melanocortin 3* (*MC3R*) gene expression to identify new biomarkers inflammation in tuberculosis

**DOI:** 10.1186/s43042-022-00323-8

**Published:** 2022-07-18

**Authors:** Andi Tenriola, Najdah Hidayah, Subair, Muhammad Nasrum Massi, Handayani Halik, Tri Damayanti, Jafriati, Andi Tenri Ola Rivai

**Affiliations:** 1 Postgraduate Program, Faculty of Medicine, Universitas Hasanuddin, Jl. Perintis Kemerdekaan Km.10, Makassar, Sulawesi Selatan 90245 Indonesia; 2 Department of Microbiology, Faculty of Medicine, Universitas Hasanuddin, Jl. Perintis Kemerdekaan Km.10, Makassar, Sulawesi Selatan 90245 Indonesia; 3 Department of Public Health Study Program, Faculty of Public Health, Universitas Halu Oleo, Kampus Hijau Bumi Tridharma, Anduonohu, Kec. Kambu, Kota Kendari, Sulawesi Tenggara 93232 Indonesia; 4 Universitas Islam Negeri Alauddin, Makassar, Jl. Sultan Alauddin No. 63, Romangpolong, Kec. Somba Opu, Kabupaten Gowa, Sulawesi Selatan 92113 Indonesia

**Keywords:** *Melanocortin 3*, *MC3R*, Gene expression, TB, Tuberculosis

## Abstract

**Background:**

Diagnosis of tuberculosis (TB) in the era of technological sophistication requires accuracy and speed to provide as much information as possible so that TB treatment can be carried out quickly and precisely. New studies have also begun to be carried out to diagnose TB, one of which is by examining genes, either by looking at polymorphisms, mutations, or expressions. Several previous studies have confirmed the association of *MC3R* and TB genes with polymorphisms; *MC3R* is a gene that participates in the regulation of the inflammatory process and is also found in macrophages; therefore, we tried to analyze gene expression in the active TB group, household contacts, and healthy controls for looked at the differences between the three groups and confirmed the correlation of *MC3R* with TB by seeing which group's gene expression increased the most expression of the three groups so that the results can be considered as a TB diagnostic biomarker in the future.

**Methods:**

This study included 122 people, 49 patients with confirmed TB, 46 close relatives of patients, and 27 healthy controls. This study used a real-time PCR technique to analyze *MC3R* gene expression in the three groups, and all data were analyzed using Bio-Rad CFXTM software version 3.1 and one-way ANOVA using SPSS 21.0.

**Results:**

The value of *MC3R* gene expression in the active TB group increased 3.6-fold in the healthy group (*p* = 0.143), and that of gene expression in the healthy control group increased 1.09-fold in the healthy group (*p* = 0.007).

**Conclusion:**

There is a relationship between *MC3R* and TB based on the results of gene expression analysis that increased in the active TB group compared to the household contact group and healthy controls.

## Background

The number of individuals living with tuberculosis in 2020 is over multiple times the assessed yearly frequency of tuberculosis. WHO areas in Southeast Asia (44%), Africa (25%), and the Western Pacific (18%) have the most TB victims. Eight nations represent 66% of the worldwide aggregate: India (26%), Indonesia (8.5%), China (8.4%), Philippines (6.0%), Pakistan (5.7%), Nigeria (4 0.4%), Bangladesh (3.6%), and South Africa (3.6%) [1]. By 2020, the COVID-19 pandemic will dislodge TB from the irresistible infection driving the reasons for death internationally [2]. Worldwide, TB control endeavors did not work out as expected even before the development of the COVID-19 pandemic [3]. There are as yet many difficulties in further developing TB treatment and counteraction benefits that are not exactly ideal [4]. Tuberculosis screening and analytical testing administration should be helped; delays in determination and consistent transmission add to the high death rate in Indonesia, which has the third most elevated TB occurrence on the planet, after China and India [5]. Consequently, an early determination is expected to diminish the number of cases and give treatment to forestall bacterial transmission [6]. Finding TB stays a test in clinical practice because of helpless affectability and the requirement for talented staff in tiny testing, slow development of Mycobacterium [7].

*MC3R (MC3, Melanocortin-3)* is a gene that participates in the regulation of circadian rhythm activities associated with eating behavior and anti-inflammatory processes [8]. Studies have shown that *MC3R* may be involved in the development of inflammatory diseases, such as pulmonary tuberculosis and arthritis [9]. The health conditions associated with *MC3R* genetic alterations are quantitative and lead to susceptibility to Mycobacterium tuberculosis [10]. Several previous studies in several populations have shown that genetic factors contribute to tuberculosis, with estimates of heritability ranging from 36 to 80% [11]. In the journal Association of *CTSZ* rs34069356 and *MC3R* rs6127698 Gene Polymorphisms with Pulmonary Tuberculosis, it was stated that *MC3R* is a receptor that is widely expressed in the brain and various peripheral tissues and has been shown to contribute to many biological systems including inflammation and infection [12].

Early conclusion of TB is vital to forestall its spread; the corrosive quick sputum smear test, albeit speedy and reasonable, is not the most delicate and explicit indicative test [13]. While the tuberculin skin test is a typical symptomatic technique, it tends to deliver bogus positive outcomes in people recently vaccinated with BCG [14]. The culture of TB microscopic organisms typically sets aside time and determination dependent on test results is not generally exact. Interferon gamma discharge examiners (IGRA) seem to be the best quality level for TB testing [15]. The test has been acquainted in clinical practice with the measure of interferon gamma (IFN-γ) delivered by Mtb-contaminated platelets; lamentably, this strategy is more costly and requires a blood test to a typical leukocyte level [16], which is not generally imaginable in immunocompromised people. Accordingly, elective quantitative polymerase chain response techniques were created to recognize the safe reaction to TB contamination [17]. Be that as it may, as most quality articulation concentrates to show, the hereditary foundations can impact the particularity and affectability of the conclusion [18].

*MC3R* gene expression and *MC3R* protein levels may in the future become new biomarker candidates for diagnosing active TB by the real-time PCR because the real-time PCR is on real time of the most widely used techniques in modern molecular biology to see amplification quickly, and even this technique can be used to determine the concentration of DNA contained in the sample. When compared with other types of diagnostic tests for the diagnosis of TB, this *MC3R* gene expression test can show the genetic contribution of TB in a larger population [19].

The World Health Organization (WHO) has designated a 90% decrease in the frequency of tuberculosis (TB) and a 95% decrease in TB-related passing by 2035 [20]. The current pace of decrease is inadequate to accomplish this objective, and commonness studies in a few nations have shown an enormous weight of TB [21], undiscovered locally. Subsequently, WHO currently suggests deliberate evaluation for TB in high-hazard gatherings, one of which is quality articulation [22]. Right and exact distinguishing proof of causative specialists like organisms in microbial infections, explicit hereditary succession in hereditary illnesses, and protein levels are fundamental for the administration of these patients, making explicitness and affectability a significant apparatus in the analysis [23]. Traditional atomic procedures, for example, ordinary PCR and blotching, however, assume a good part in the finding [24].

In 2011, Lu et al. led a quality articulation microarray study to explore the chance of utilizing mRNA as a biomarker to separate dynamic TB from LTBI. Curiously, in their review, the declaration of IFN-γ, a biomarker utilized in IGRAs, did not vary fundamentally between the dynamic TB and LTBI gatherings. Interestingly, the mix of three qualities, CXCL10 (chemokine CXC theme ligand 10), ATP10A (ATPase, class V, type 10A), and TLR6 (cost-like receptor 6), gave off an impression of being successful in separating among dynamic and inert TB contamination. Interestingly, IL-8 (Interleukin 8), FOXP3 (forkhead box P3), and IL-12β (interleukin 12 beta) were demonstrated to be the best-separating biomarkers for TB and LTBI by Lu et al. [25]. Therefore, we are interested in looking at the results of the examination of *MC3R* gene expression using the real-time PCR method to see whether the examination of gene expression using the real-time PCR method can be one of the diagnostic options for TB in the future.

## Methods

A sum of 122 examples from the age of 17–70 years was gathered and isolated into three gatherings: active TB, family contacts, and solid. Forty-nine dynamic TB patients were affirmed to go through treatment at the Makassar Community Lung Health Center (BBKPM), 46 family contacts who had a connection with dynamic TB patients had interferon gamma release assay (IGRA) assessments performed, and 27 sound individuals did not show the manifestation of TB and negative IGRA results. This review was endorsed by the Research Ethics Commission of the Faculty of Medicine, Hasanuddin University, Makassar, South Sulawesi, Indonesia (No. 517/H4.8.4.5.31/PP36-KOMETIK/2018 on 27 July 2018), and assent was taken from all patients. All examinations were done keeping important rules and guidelines and done in a laboratory HUM-RC (Hasanuddin University Medical Research Center) Makassar, Indonesia.

### RNA extraction

The initial phase in inspecting the declaration of this quality is to extricate RNA from the gathered blood tests, then, at that point, lyse the cells by adding 400uL of RB buffer and 4uL of mercaptoethanol and afterward hatched for 3 min at room temperature. A channel section with a 2-mL assortment tube was introduced. The whole blend was moved to the channel section and afterward centrifuged 1000× g for 30 s. The channel section was cautiously moved into another 1.5-mL Eppendorf tube. The following stage is RNA-restricted by adding 400 L of 70% ethanol arranged with ddH2O into the cylinder and afterward moving the whole combination into the RB segment (introduce the RB segment with a 2-mL collection tube first). Axis 14–16.000× g for brief, then the supernatant was taken out and reassembled with the RB section. The following stage in this stage is washing, where, however, much 400 L of W1 buffer is added to the RB column and afterward centrifuged 14–16,000 xg for 1 moment, dispose of and reinstall the RB column, and then, at that point, add 600 L wash buffer (make sure the wash buffer has been added to ethanol) into the RB segment. Rotator was centrifuged 14–16,000× g for 30 s and then, at that point, disposed of and reinstalled the RB section. A sum of 600 L wash buffer was added to the RB column and then centrifuged 14–16,000× g for 30 s. The RB segment was disposed of and reinstalled and afterward centrifuged again 14–16.000× g for 3 min to dry the network section. The last stage was RNA development, the RB column was moved to another 1.5-mL Eppendorf tube (clean), then, at that point, 50 L of RNase-free water (to expand the RNA focus, hatched at room temperature for 1 moment) was added, and then, at that point, centrifuged 14–16,000× g for 1 moment. 1000× g for 30 s then, at that point, eliminate and reinstall the RB section. An aggregate of 600 L wash buffer was added to the RB column and then centrifuged 14–16,000× g for 30 s. The RB segment was disposed of and reinstalled and afterward centrifuged again 14–16.000× g for 3 min to dry the network segment. The last stage was RNA development, the RB column was moved to another 1.5-mL Eppendorf tube (clean), then, at that point, 50 L of RNase-free water (to expand the RNA fixation, brooded at room temperature for 1 moment) was added, and then, at that point, centrifuged 14–16,000× g for 1 moment. 1000× g for 30 s then, at that point, eliminate and reinstall the RB section. A sum of 600 L wash buffer was added to the RB column and then centrifuged 14–16,000× g for 30 s. The RB segment was disposed of and reinstalled and afterward centrifuged again 14–16.000× g for 3 min to dry the grid segment. The last stage was RNA development; the RB column was moved to another 1.5-mL Eppendorf tube (clean) and then, at that point, added 50 L of RNase-free water (to expand the RNA fixation, brooded at room temperature for 1 moment) and then, at that point, centrifuged 14–16,000× g for 1 moment.

### Reciprocal DNA (cDNA) intensification with reverse transcriptase-PCR

The intensification of cDNA from RNA extraction utilizing RT PCR was done which is dependent on the Invitrogen strategy. The technique was done utilizing the SuperScript First-Strand Synthesis System for RT-PCR (Invitrogen). The expert blend I was made by adding 5 of RNAμg all-out RNA, 3 μl irregular hexamers (50 ng/μl), 1 μl 10 mM dNTP blend, and nuclear-free water (H2O) so the complete PCR volume is 50 μl. The examples were then hatched at 65 °C for 5 min and afterward on ice for no less than 1 moment. Then, the expert blend II response combination was ready by adding 2 μl 10 × RT cushion, 4 μl, 25 mM MgCl2, 2 μl 0.1 M DTT, and 1 μI RNAase out. Add the response blend to the RNA/essential combination blend momentarily, and then, at that point, place at room temperature for 2 min. Add 1 μl (50 units) SuperScript II RT into each cylinder blend and hatch at 25 °C for 10 min, then, at that point, brooded at 42 °C for 50 min, for inactivation warmed at 70 °C for 15 min, and afterward cooled on ice. Next, add 1 μl RNase H and hatched at 37 °C for 20 min. cDNA strands can be put away at − 20 °C until utilized for continuous PCR.

### Assessment of MC3R quality articulation with ongoing PCR

Assessment of the strength of quality articulation (up-guideline or down-guideline) of *MC3R* utilizing SYBR green color with ongoing PCR (qPCR) technique. Before intensification, an expert blend was ready by adding 12.5 μl SYBR green blend, 0.5 μl cDNA, 0.5 μl groundwork forward (CAACACTGCCTAATGGCTCGGA), 0.5 μl turnaround preliminaries (GTTTTCCAGCAGACTGACGATGC) (5 pmol/each primer μl), and 11.3 μl H2O. A similar method was likewise performed for the GAPDH quality as a control, yet utilizing an alternate groundwork arrangement. GAPDH forward groundwork (CAACACTGCCTAATGGCTCGGA) and converse GAPDH preliminary (GTTTTCCAGCAGACTGACGATGC) cycle qPCR under the states of 50 °C for 2 min, 95 °C for 1 moment for 1 cycle every, denaturation at 95 °C for 15 s, and then, at that point, trailed by tempering at 60 °C for 30 s and augmentation at 72 °C for 30 s rehashed multiple times (cycles); the last cycle is the last expansion at 72 °C for 10 min.

## Results

An aggregate of 122 examples of different sexual orientations and ages were remembered for this review. In dynamic TB, there were 20 (41%) men and 29 (59%) ladies. 8 (16.3%) individuals matured 17–29 years, 27 (55.1%) individuals matured 30–49 years, and 14 (28.5%) individuals matured 50–70 years. In the family contact bunch, there were 18 (39%) men and 28 (61%) ladies. In view of old enough, 9 (19.6%) individuals matured 17–29 years, 14 (30.4%) individuals matured 30–49 years, and 23 (half) individuals matured 50–70 years. At long last, in the solid benchmark group, there were 10 (37%) male examples and 17 (63%) female examples. In view of old enough rules, in this gathering, there are 5 (18.5%) individuals matured 17–29 years, 11 (40.7%) individuals matured 30–49 years, and 11 (40.7%) individuals matured 50–70 years as we can see in Table 1.

**Table 1:** Characteristics of the sample based on the active TB group, household contacts, and healthy controls

	Active TB	Household contact	Healthy participants	Total
	*n* = 49	*n* = 46	*n* = 27	
*Gender*				
Male	20 (41%)	18 (39%)	10 (37%)	48
Female	29 (59%)	28 (61%)	17 (63%)	74
*Age*				
17–29 years	8 (16.3%)	9 (19.6%)	5 (18.5%)	22
30–49 years	27 (55.1%)	14 (30.4%)	11 (40.7%)	52
50–70 years	14 (28.5%)	23 (50%)	11 (40.7%)	48

**Fig. 1:**
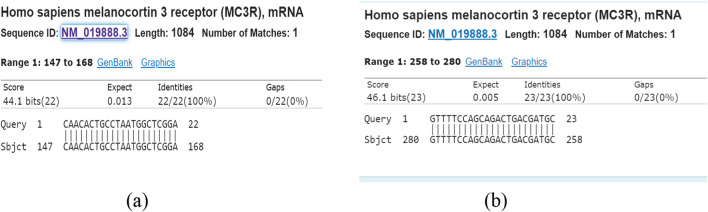
**a** *MC3R* primer forward (CAACACTGCCTAATGGCTCGGA); **b** *MC3R* primer reverse (GTTTTCCAGCAGACTGACGATGC)

In view of the aftereffects of quality articulation investigation in this review, there was a considerable expansion in *MC3R* quality articulation in the dynamic TB bunch (*p* = 0.143). The articulation worth can be found in Table 2 and Fig. 1. Although there have been no past investigations examining the connection between expanded *MC3R* quality articulation and TB, we affirm a potential connection between *Melanocortin 3* receptors. An investigation of *MC3R* polymorphism in a Han populace in Southern China (2020) examined the relationship between the two with 341 TB patients (173 with aspiratory TB and 168 with multifocal TB) and 359 sound controls. The finish of this review affirmed an expansion in protein articulation in patients with multifocal TB contrasted with solid controls (*p* < 0.05) [20]. One more review directed by Lindsey Adams et al. (2011) entitled polymorphisms in *MC3R* advertisers and *CTSZ* 3'UTR are related to tuberculosis powerlessness has approved the relationship of two qualities (*CTSZ* and *MC3R*) with tuberculosis vulnerability, a further examination concerning the advancement of TB [26, 27].

**Table 2:** Gene expression values in active TB, household contact, and healthy controls

Target	Biological group	Control	Expression	Expression 95% CI low	Expression 95% CI high	*p value*
GAPDH	Active TB					
	Household control					
	Health	C				
*MC3R*	Active TB		3.1548	1.3473	7.3872	0.143
	Household control		0.9523	0.5518	1.6437	0.007
	Health	C	0.8678	0.5817	1.2945

**Fig. 2:**
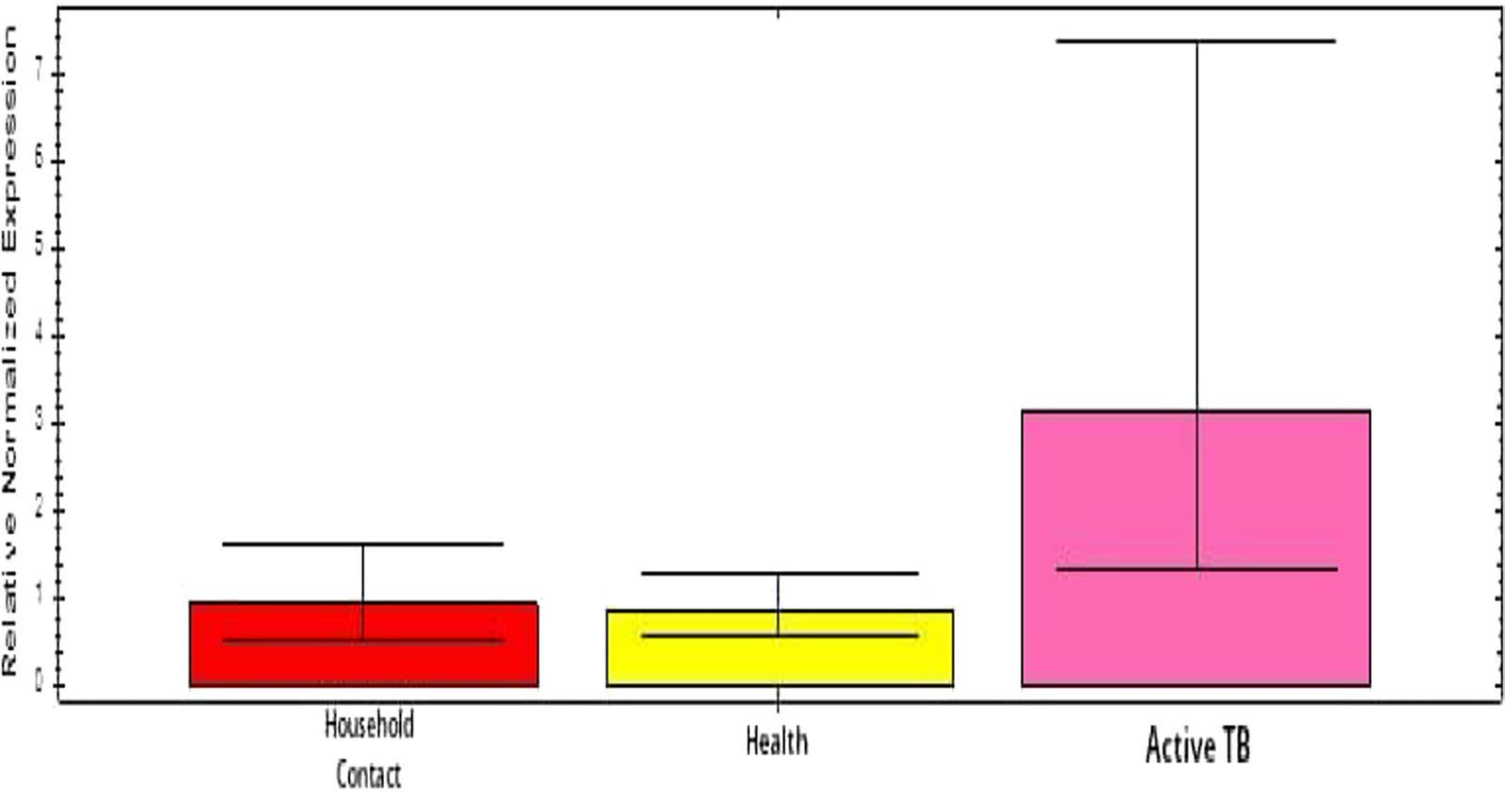
Bar chart results of gene expression analysis in the active TB group, household contact, and healthy controls

Table 2 and Fig. 2 are the results of gene expression analysis showing the results of the examination of *MC3R* gene expression in the three groups of research samples. The results of the analysis of *MC3R* gene mRNA expression based on the cq value using the Livak Eq. (2–∆∆Cq) showed that the gene expression value of the active TB group increased 3 times compared to the healthy TB group and the contact group and was significantly different with the *p* value = 0.007 (see Table 2 and Fig. 2). This also explains the association between increased *MC3R* gene expression and active TB.

### MC3R expression in active TB patients compared to positive and negative IGRA participants (negative IGRA household contacts and healthy group)

**Fig. 3:**
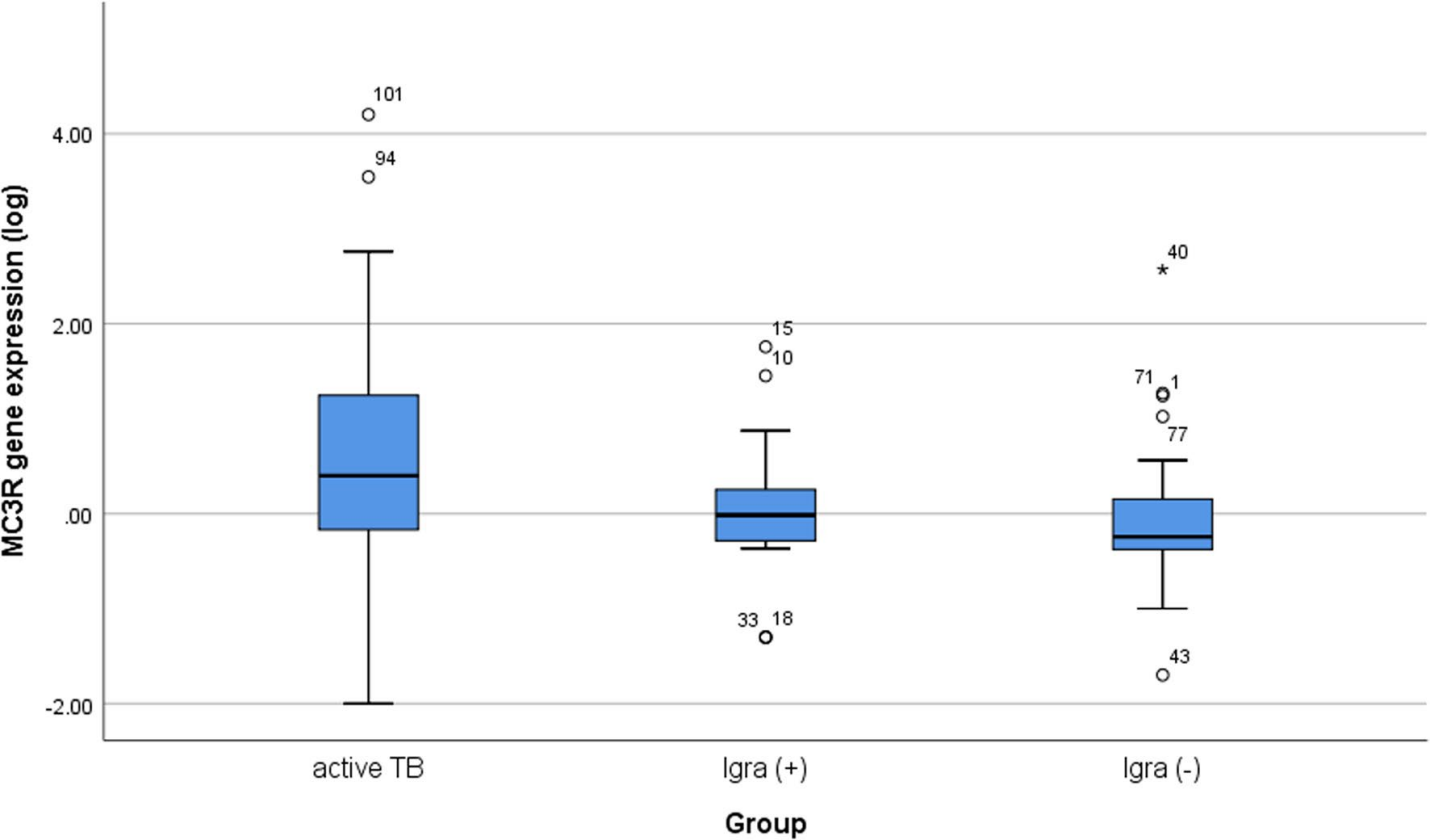
Kruskal–Wallis test

Based on the Kruskal–Wallis test, *MC3R* gene expression was higher in active TB patients than in positive and negative IGRA participants (negative IGRA household contacts and healthy group) (*p* = 0.004), as shown in Fig. 3

## Discussion

**Fig. 4:**
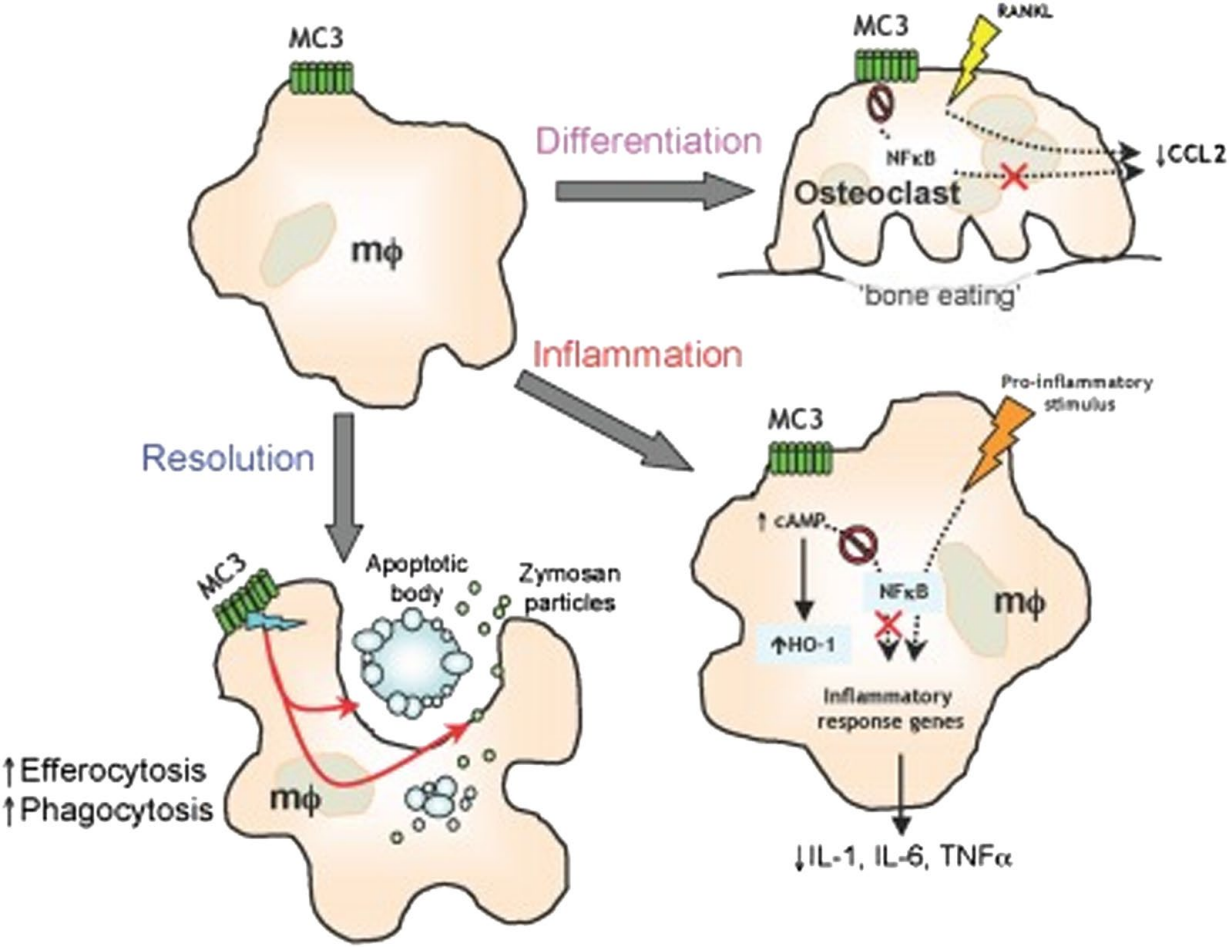
Type 3 melanocortin receptor activation on macrophage function MC3 activation by endogenous or selective synthetic agonists leads to regulation of osteoclast generation, control of pro-inflammatory and anti-inflammatory mediators, and resolution of inflammation by spherocytosis and phagocytosis

Increased expression of the *MC3R* gene and high levels of *MC3R* protein in active tuberculosis is associated with its physiological role as a regulator of energy metabolism and the immune system. In its role in regulating physiological responses, *MC3R* is associated with immunity, where one of the melanocortin peptides that play a role in immune activity is -MSH and ACTH [27]. *MC3R* activation by -MSH and ACTH causes the secretion of the anti-inflammatory cytokine interleukin 10 (IL-10) [28]. In disease pathophysiological processes, -MSH suppresses inflammation and promotes activation of inflammatory activity and immune regulation through *MC1R*, *MC3R*, and *MC5R*, whereas in macrophages, -MSH mediates alternative activation where macrophages suppress inflammation through *MC1R* and *MC3R* [29]. Macrophages are the body's defense, which is part of the innate immune system that gives the body the ability to destroy invading mycobacteria. In the process of the pathogenesis of tuberculosis, the war between Mycobacterium and macrophages will result in two possibilities: if macrophage cells are strong, they will destroy Mtb, but if macrophages are weak, Mtb will continue to replicate in macrophage cells; the body's specific immune system will continue to work against Mtb. Macrophages will surround Mtb and form multinucleated cells, this is what will trigger an increase in melanocortin levels including *MC3R* which plays an important role in promoting anti-inflammatory activity and regulating the body's immune system against Mtb, as shown in Fig. 4 [30]. Although the pathogen has a remarkable capacity to survive in a hostile macrophage environment, the primary infection does not cause active TB disease in most people even those who have been infected latently [31]. The majority of individuals remain infected latently, in which the bacteria are controlled by the host's immune response.

Albeit this review is another review that talks about the statement of the *MC3R* quality in the TB bunch, and the set number of tests is still moderately little, this review gives an outline of the up-guideline of quality articulation in dynamic TB, just as contrasts in articulation in the other two gatherings, specifically family contacts and sound controls. The outflow of the *MC3R* quality may in the future become a new biomarker for diagnosing dynamic TB by the constant PCR strategy, since continuous PCR is one of the most generally utilized methods in current atomic science to see intensification rapidly, and this procedure can likewise be utilized to decide the centralization of DNA present in the example [32]. In past examinations, *MC3R* was related to TB susceptibility, macrophages interceding the host's intrinsic invulnerable reaction against Mycobacterium tuberculosis through microbe acknowledgment and initiation of the fiery reaction (33). Mycobacterium tuberculosis lives in the phagolysosomes of macrophages, where it avoids the resistant reaction in most contaminated people [34]. In dynamic TB, macrophages neglect to restrain bacillary replication, coming about in the expanded *MC3R* articulation [35]. We presume that this diverse macrophage reaction to Mycobacterium tuberculosis is related to the hereditarily unique articulation brought about by these three gatherings [36].

When contrasted with different sorts of analytic tests for the analysis of TB, this *MC3R* quality articulation test can show the hereditary commitment of TB in a bigger populace, since this *MC3R* is a receptor that is generally communicated in the cerebrum and different fringe tissues and has been displayed to contribute a great deal to organic frameworks, particularly in energy homeostasis, invulnerability and irritation, so the outcomes are more exact when used to plan the hereditary qualities of TB in bigger examples or populaces [32, 37].

The results of our study are expected to add references to the basic understanding of immunity against TB which will later be useful for the development of TB diagnosis and therapy. Basic issues regarding TB are not only related to the duration of TB diagnosis, but also the development of an effective TB vaccine into adulthood [38], plus the widespread cases of drug resistance to potent oats such as isoniazid and rifampin, known as MDR [39]. With a better understanding of the host's immune response to TB infection, the development of a fast and accurate diagnosis, investigation of biomarkers to support the diagnosis, and research on immunotherapy as an alternative to oat treatment from the rise of resistance cases can be more focused [40].

## Conclusion

This study includes a new study that discusses *MC3R* gene expression in the TB group; although the limited number of samples is still relatively small, this study has provided an overview of the increased *MC3R* gene expression in the active TB group compared to household contacts and healthy controls that may be possible, which will be investigated further by future researchers. Expression of the *MC3R* gene may in the future become a new biomarker for diagnosing increased inflammatory reactions in active TB by the real-time PCR method, because real-time PCR is one of the most widely used techniques in modern molecular biology to see amplification quickly, and this can also be used to determine the concentration of DNA contained in the sample. When compared with other types of diagnostic tests for the diagnosis of TB, this *MC3R* gene expression test can show the genetic contribution of TB in a larger population, because this *MC3R* is a receptor that is widely expressed in the brain and various peripheral tissues and has been shown to contribute to many biological systems, especially in energy homeostasis, immunity, and inflammation, so the results are more accurate when used to map the genetics of TB in larger samples or populations.

## Data Availability

Data are available from the authors upon reasonable request.

## Abbreviations

ACTH: Adrenocorticotropic hormone
BBKPM: Balai Besar Kesehatan Paru Masyarakat
BCG: Bacillus Calmette Guerin
cDNA: Complementary DNA
CTSZ: Cathepsin Z
DNA: Deoxyribonucleic acid
GAPDH: Glyceraldehyde 3-phosphate dehydrogenase
H2O: Dihydrogen monoxide
IGRA: Interferon gamma release assay
MC3: Melanocortin 3
MCR: Melanocortin receptor
MC1R: Melanocortin 1 receptor
MC3R: Melanocortin 3 receptor
MC5R: Melanocortin 5 receptor
mRNA: Messenger RNA
Mtb: Mycobacterium tuberculosis
MSH: Melanocortin-stimulating hormone
PCR: Polymerase chain reaction
qPCR: Quantitative polymerase chain reaction
RNA: Ribonucleic acid
RT-PCR: Real-time polymerase chain reaction
TB: Tuberculosis
WHO: World Health Organization
